# Bioinformatics and *In Silico* Findings Uncover Bio-Targets of Calycosin Against Heart Failure and Diabetes Mellitus

**DOI:** 10.3389/fendo.2022.790619

**Published:** 2022-07-08

**Authors:** Hongyuan Xu, Jingru Qin, Lixiu Qin, Chao Guo, Bin Yang

**Affiliations:** ^1^ Cardiology Department, Guigang City People’s Hospital, The Eighth Affiliated Hospital of Guangxi Medical University, Guigang, China; ^2^ College of Pharmacy, Guangxi Medical University, Nanning, China; ^3^ Department of Pharmacy, Guigang City People’s Hospital, The Eighth Affiliated Hospital of Guangxi Medical University, Guigang, China

**Keywords:** heart failure, diabetes mellitus, calycosin, target, mechanism, network pharmacology

## Abstract

**Background:**

Heart failure (HF) and diabetes mellitus (DM) are life-threatening diseases. However, existing clinical drugs to treat HF complicated with DM are relatively limited. In this study, we performed a viable bioinformatics strategy combining network pharmacology and molecular docking to identify potential anti-HF and -DM targets and therapeutic mechanisms of calycosin, a functional phytoestrogen.

**Methods:**

Web-based databases were used to collect candidate genes/targets of calycosin and HF/DM and then identify the hub bio-targets of calycosin against HF/DM. Using the online-available database, all functional processes and signaling pathways of calycosin against HF/DM were screened and identified before further visualization.

**Results:**

All potential bio-targets of calycosin and HF/DM were collected, and 20 hub targets of calycosin against HF/DM were identified. Interestingly, molecular docking findings indicated that mitogen-activated protein kinase-1 (MAPK1), β-arrestin 1 (ARRB1), and homologue-1 (ABL1) may be potent pharmacological targets of calycosin against HF/DM. In addition, all primary molecular functions of calycosin against HF/DM were identified, including regulating protein binding, ubiquitination, and the metabolic process. Furthermore, the top molecular pathways of calycosin against HF/DM were revealed, including cardiomyocyte and chemokine signaling pathways.

**Conclusion:**

Our bioinformatics analysis uncovered the network targets and therapeutic mechanisms of calycosin against HF/DM. For the first time, the current *in silico* findings revealed that the identified hub targets may be used to screen and treat HF/DM.

## Background

Heart failure (HF) refers to the inability of the heart to circulate sufficient blood to sustain normal functions in the body (1). As an emerging health issue, the annual cases of HF are increasing worldwide (2). In China, a rising prevalence of HF is reported, presenting an economic burden (3). Clinically, patients with HF show visible symptoms of lethargy, dizziness, and dyspnea, and the risk factors include aging, hypertension, and chronic metabolic disorders (4). In acute HF, the medical treatment includes oxygen supply, inotropic agents, and other supportive therapies (5). The common treatment for chronic HF involves prescribing neuroendocrine inhibitors as long-term restorative strategies (6). Diabetes mellitus (DM), characterized by hyperglycemia, is caused by a relative or absolute deficiency of insulin in the body (7). In China, DM cases have been increasing in recent years, especially in the young population (8). Clinically, patients with DM may have an increased risk of developing HF, and thus, the therapeutic effectiveness and prognosis of HF associated with DM are poor (9). In addition, some adverse effects of chemotherapeutic-based therapy for HF/DM are reported in clinical practice. In further investigations, potential pharmacologically effective components for managing HF/DM will be screened and identified. In China, Radix Astragali is traditionally used in treating chronic HF (10). Calycosin, a functional phytoestrogen chemically extracted from *Radix Astragali*, has potent pharmacological actions, such as anticancer, neuroprotection, cytoprotection, and anti-diabetes activity (11–13). However, reports regarding calycosin-exerted anti-HF/-DM activity are lacking. Along with the development of modern bioinformatics, systematic pharmacology is currently used for identifying the candidate targets and therapeutic pathways of existing agents for treating clinical diseases (14, 15). Interestingly, our previous study showed the bioinformatic findings of vitamin C effects against liver injury and revealed the therapeutic targets and biological mechanisms for perfluorooctanesulfonate-associated leukemia (16, 17). As the anti-HF/-DM mechanisms remain uninvestigated, we used an effective strategy combining network pharmacology and molecular docking to identify the functional bio-targets and therapeutic mechanism of calycosin effects against HF/DM.

## Materials and Methods

### Detection of Candidate Genes/Targets

Preliminary data were collected from the PubMed database. Retrieval keywords were subjected with “calycosin,” “heart failure, and “ “diabetes mellitus” (MeSH) terms. In addition, literatures were restricted to the journal original paper and English language. The databases Traditional Chinese Medicine Systems Pharmacology Database and Analysis Platform (TCMSP), Chemical-Protein Interaction Networks (STITCH; http://stitch.embl.de/), Swiss Target Prediction, SuperPred webserver, Bioinformatics Analysis Tool for Molecular mechANism of Traditional Chinese Medicine (BATMAN-TCM; http://bionet.ncpsb.org/batman-tcm/), ChemMapper, and Drug Target Prediction System were collectively used to screen the functional genes/targets of calycosin. Furthermore, the HF- and DM-driven genes/targets were acquired from the detection databases DisGeNET, GeneCard, DrugBank (www.drugbank.ca), PharmDB-K, and Therapeutic Target Database (TTD). After mapping the genes/targets of calycosin and HF/DM using an online website (http://bioinformatics.psb.ugent.be/webtools/Venn/), the predictive targets of calycosin against HF were determined (18, 19).

### Screening Hub Targets and Constructing Protein–Protein Interaction Network

In brief, all mapped predictive targets were further analyzed after creating a target–target-associated functional interprotein network and protein–protein interaction (PPI) network using TSV data from the STRING database. The final hub targets of calycosin against HF/DM were obtained using the Network Analyzer tool in Cytoscape (v3.7.1) software, calculating from the degree values of topology parameters (20, 21).

### Molecular Docking Analysis

The molecular structure of the niacin compound was obtained from the PubChem database (https://pubchem.ncbi.nlm.nih.gov/). The protein structures of HF/DM targets were obtained from the Protein Data Bank database (https://www.rcsb.org/). Molecular mechanics-2 force field optimization was conducted using the three-dimensional structure of the compound downloaded using the ChemBio3D Draw module in the ChemBioOffice software (version 2010). The pdbqt structure file necessary for virtual screening was created using the Raccoon software. Next, the docking active center (including all residues around the original ligand) was set using the grid box function in the software, according to the root mean square deviation (RMSD) between the docked ligand and original ligand molecules with appropriate docking parameters. RMSD ≤4 is generally believed to be the threshold for the ligand conformation after docking to match the original ligand conformation (22, 23).

### Gene Ontology Biological Processes and Kyoto Encyclopedia of Genes and Genomes Enrichment Analyses

Briefly, the ClusterProfiler, ReactomePA, and AnnotationHub packages in R software (3.6.1) were used to perform a gene ontology (GO)–based biological process and Kyoto Encyclopedia of Genes and Genomes (KEGG) pathway enrichment analyses of hub targets. Finally, the bubble charts, graphs, and histograms were obtained and visualized (24, 25).

### Visualization of Network Relationships

Furthermore, using the Cytoscape software (https://cytoscape.org/), the data of hub targets in the biological processes and pathways of calycosin against HF/DM were used to construct drug–target–GO and function–pathway–disease visualization graphics (26, 27).

## Results

### Candidate and Functional Genes

**Figure 1:**
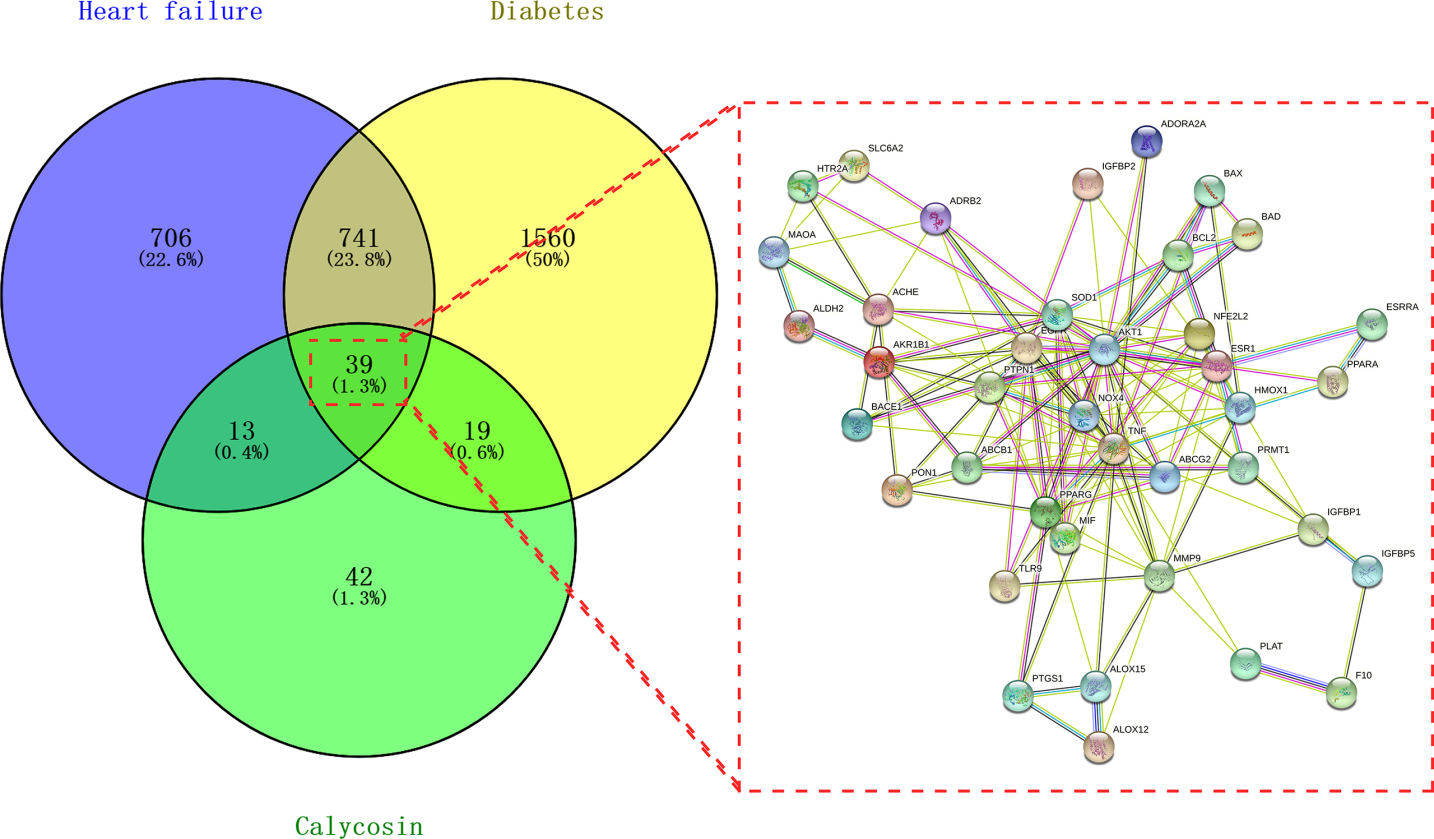
Venn diagram of calycosin effects against heart failure (HF) and diabetes mellitus (DM). Mapped targets of calycosin and HF/DM for constructing a protein–protein interaction network.

Following the bioinformatics analysis, the accessible data revealed 1,499 and 2,359 HF- and DM-related genes, respectively. Furthermore, 113 genes of calycosin were found after screening and removing duplicates using the UniProt database (https://www.uniprot.org/). As shown in **Figure 1**, 39 mapped targets are displayed in a Venn diagram. Furthermore, these PPI data with 39 targets were collected for constructing a target–target functional interaction network.

### Protein–Protein Interaction Topology Parameters and Hub Targets

**Figure 2:**
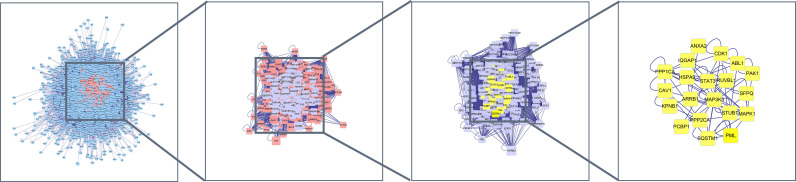
Hub targets of most important molecules of calycosin against HF and DM.

The mapped intersection proteins were imported into Cytoscape software to determine the topological parameters of calycosin against HF/DM. Screening parameters included betweenness, closeness, and connectivity, and the yellow node represented the final screening result. Twenty hub targets highly correlated with calycosin against HF/DM were obtained. These included mitogen-activated protein kinase-1 (MAPK1), ARRB1, ABL1, CDK1, MAP3K3, STUB1, PPP1CA, STAT3, PML, IQGAP1, HSPA9, CAV1, KPNB1, SQSTM1, RUVBL1, SFPQ, PPP2CA, PCBP1, ANXA2, and PAK1 (**Figure 2**).

### *G*ene Ontology Biological Processes and Kyoto Encyclopedia of Genes and Genomes Pathways Involving Hub Targets

**Figure 3:**
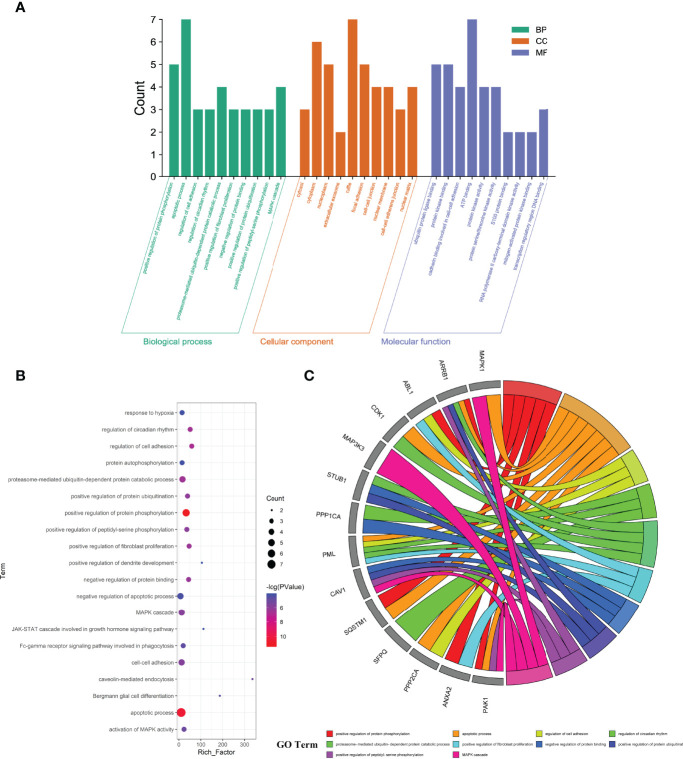
Key functional and biological functions of calycosin against HF and DM, characterized in bar diagram **(A)**, bubble chart **(B)**, and circle diagram **(C)**.

**Figure 4:**
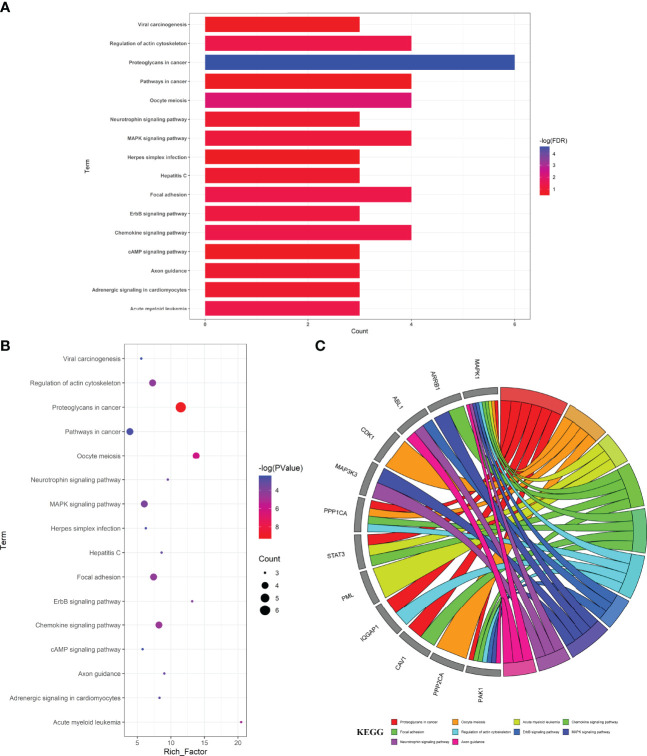
Top target–pathway associations for anti-HF/-DM activity exerted by calycosin associated with hub targets, characterized in bar diagram **(A)**, bubble chart **(B)**, and circle diagram **(C)**.

**Figure 5:**
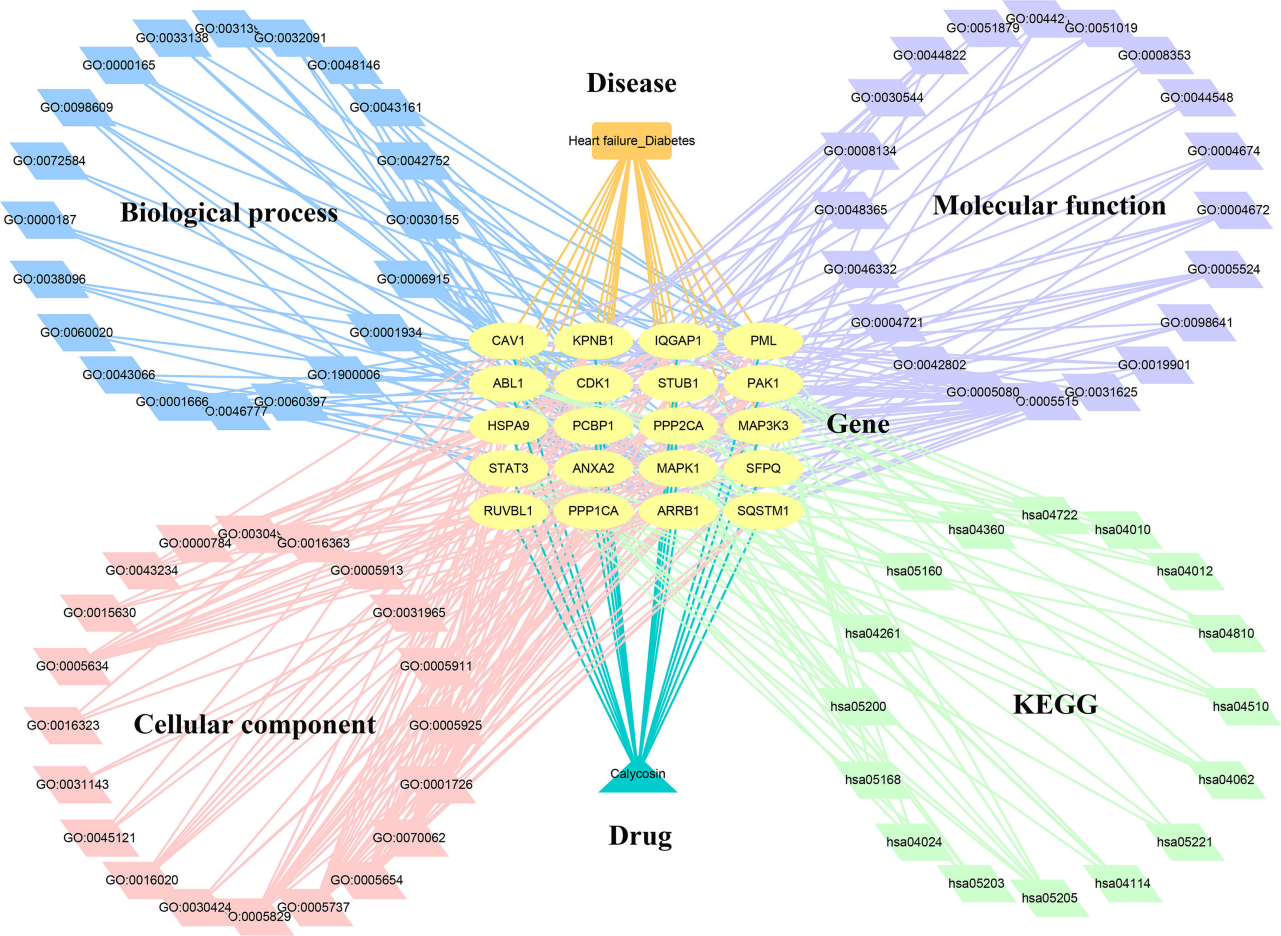
Interaction network of calycosin–target–gene ontology– Kyoto Encyclopedia of Genes and Genomes –HF/DM.

The enrichment analyses showed that the main GO-based biological processes of hub targets were involved in the positive regulation of protein phosphorylation, apoptotic process, regulation of cell adhesion, regulation of circadian rhythm, proteasome-mediated ubiquitin-dependent protein catabolic process, positive regulation of fibroblast proliferation, negative regulation of protein binding, positive regulation of protein ubiquitination, positive regulation of peptidyl-serine phosphorylation, MAPK cascade, cell–cell adhesion, caveolin-mediated endocytosis, activation of MAPK activity, Fc-gamma receptor signaling pathway involved in phagocytosis, Bergmann glial cell differentiation, negative regulation of the apoptotic process, response to hypoxia, protein autophosphorylation, Janus Kinase (JAK)-Signal Transducer and Activator of Transcription (STAT) cascade involved in the growth hormone signaling pathway, and positive regulation of dendrite development. The cellular components (CCs) of hub targets were related to cytosol, cytoplasm, nucleoplasm, extracellular exosome, ruffle, focal adhesion, cell–cell junction, nuclear membrane, cell–cell adherens junction, nuclear matrix, midbody, nuclear chromosome, telomeric region, protein complex, microtubule cytoskeleton, nucleus, basolateral plasma membrane, pseudopodium, membrane raft, membrane, and axon. The molecular functions (MFs) of the hub targets included protein binding, ubiquitin–protein ligase binding, protein kinase binding, cadherin binding involved in cell–cell adhesion, ATP binding, protein kinase activity, protein serine/threonine kinase activity, S100 protein binding, RNA polymerase II carboxy-terminal domain kinase activity, mitogen-activated protein kinase binding, transcription regulatory region DNA binding, Hsp90 protein binding, poly(A) RNA binding, Hsp70 protein binding, transcription factor binding, Rac Guanosine Triphosphatase (GTPase) binding, Smad binding, phosphoprotein phosphatase activity, identical protein binding, and protein kinase C binding (**Figures 3A–C**). In addition, 16 KEGG pathways involving hub targets were determined (*P* < 0.05). The primary signaling pathways comprised proteoglycans in cancer, oocyte meiosis, acute myeloid leukemia, the chemokine signaling pathway, focal adhesion, regulation of actin cytoskeleton, Epidermal Growth Factor Receptor (ErbB) signaling pathway, MAPK signaling pathway, neurotrophin signaling pathway, axon guidance, hepatitis C, adrenergic signaling in cardiomyocytes, pathways in cancer, herpes simplex infection, Cyclic Adenosine Monophosphate (cAMP) signaling pathway, and viral carcinogenesis (**Figures 4A–C**). The network visualization of calycosin–target–GO–KEGG–HF/DM was achieved using Cytoscape software (**Figure 5**).

### Molecular Docking Findings

**Figure 6:**
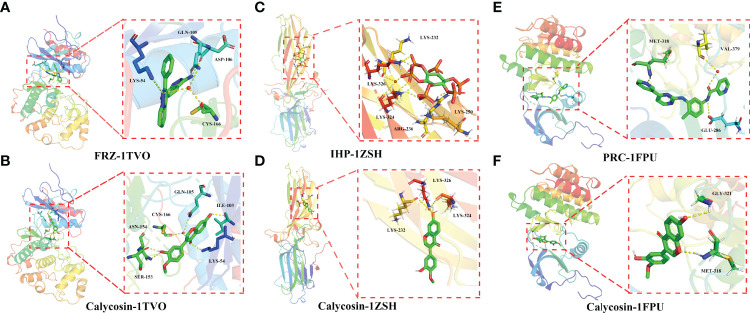
Molecular docking analysis showing the best binding activities of calycosin with 1TVO **(A)**, 1ZSH **(C)**, and 1FPU **(E)** ligands in the identified core targets of MAPK1 **(B)**, ARRB1 **(D)**, and ABL1 **(F)** in HF and DM.

As shown in **Figure 6A**, the active cavity box model of MAPK1 has parameter settings of center-x-y-z as 6.89, -3.188, and 16.85 and size-x-y-z as 15, 15, and 15, and the RMSD of the original ligand is 2.73 Å. The hydrogen bonding of the pro-ligand FRZ to the 1Tvo protein involved the amino acid residues ASP-106 (3.0Å), LYS-54 (3.5Å), GLN-105 (3.3Å), and CYS-166 (2.5 Å) and the hydrogen bond formation with calycosin included SER-153 (1.8 Å), ASN-154 (2.8Å), LYS-54 (3.0Å), ILE-103 (2.3Å), GLN-105 (3.3Å), and CYS-166 (2.5 Å) (**Figure 6B**).

As shown in **Figure 6C**, the active cavity box model of ARRB1 has center-x-y-z as -22.343, 57.812, and 3.635 and size-x-y-z as 14, 14, and 14, and the RMSD of the original ligand is 2.18 Å. Hydrogen bonding was formed between the pro-ligand IHP and 1ZSH protein through the amino acid residues LYS-326 (2.4Å), LYS-232 (3.2Å), LYS-324 (3.5Å), ARG-236 (2.0 Å), and LYS-250 (2.1 Å), and calycosin formed hydrogen bonds with LYS-326 (2.4Å), LYS-232 (3.2Å), and LYS-324 (3.5Å) (**Figure 6D**).

As shown in **Figure 6E**, the active cavity box model of ABL1 has center-x-y-z as 13.039, 95.919, and 58.059 and size-x-y-z as 30, 30, and 30, and the RMSD of the original ligand is 1.97Å. The hydrogen bonding between the pro-ligand Polycomb Repressive Complex (PRC) and 1FPU protein involved the amino acid residues MET-318 (1.9 Å), VAL-379 (3.1 Å), and GLU-286 (3.4 Å), and calycosin formed hydrogen bonds with MET-318 (2.4 Å) and GLY-321 (2.7Å) (**Figure 6F**).

## Discussion

The epidemiological data suggest that the incidence of HF is increasing due to a growing population with metabolic disorders worldwide (28). Furthermore, both HF and DM may increase mortality rates because clinical treatment using chemotherapy is medically insufficient. Hence, the pharmacological activity of potential therapeutic compounds warrants to be investigated. In the current network pharmacology and molecular docking-based approach, the candidate targets, functions, and signaling pathways of calycosin against HF/DM were identified. Furthermore, the hub bio-targets of calycosin against HF and DM were screened, namely, MAPK1, ARRB1, ABL1, CDK1, MAP3K3, STUB1, PPP1CA, STAT3, PML, IQGAP1, HSPA9, CAV1, KPNB1, SQSTM1, RUVBL1, SFPQ, PPP2CA, PCBP1, ANXA2, and PAK1. In further determination using the molecular docking analysis, the core targets of MAPK1 (1TVO), ARRB1 (1ZSH), and ABL1 (1FPU) in HF and DM showed the best binding activities with calycosin, indicating that these three genes may be potent pharmacological targets of calycosin against HF and DM. Overall, these identifiable genes may be potential therapeutic bio-targets for treating HF and DM using calycosin. MAPK1 affects translation, mitosis, and apoptosis in differentiated cells *via* phosphorylating several transcription factors (29). ARRB1, an adaptor molecule, is essential for mitogenic signaling, and its function may be induced through ERK1/2 activation (30). ABL1, a non-receptor tyrosine kinase, mainly exerts a key role in the development of solid tumors, including ovarian cancer, breast cancer, and lung cancer (31). However, there is a limited investigation of these three targets regarding calycosin action against HF and DM. Based on the present bioinformatic findings, we concluded that calycosin exerts a potential pharmacological activity to treat HF and DM, likely through regulating hub bio-target expression. In addition, some of the newly identified hub bio-targets, including MAPK1, ARRB1, and ABL1, may be promising candidates for screening the development of HF and DM. Furthermore, the top biological processes and KEGG signaling pathways of calycosin were highlighted, revealing the anti-HF/-DM pharmacological mechanisms of calycosin. These network pharmacology-based findings may immensely promote anti-HF/-DM research using calycosin clinical treatment. However, as potential limitations in the current bioinformatics report, experimentative validation *in vivo* and *in vitro* should be conducted accordingly in future studies.

## Conclusions

For the first time, using network pharmacology and a molecular docking–based approach, this study revealed the candidate hub bio-targets, biological functions, and KEGG pathways of calycosin against HF and DM. In further investigations, more validated experiments will be conducted in a preclinical study.

## Data Availability Statement

The original contributions presented in the study are included in the article/**Supplementary Material**. Further inquiries can be directed to the corresponding authors.

## Author Contributions

CG and BY: Conception and design, collection and/or assembly of data, and manuscript writing. HX, JQ, LQ: Collection and/or assembly of data and data analysis and interpretation. All authors contributed to the article and approved the submitted version.

## Funding

This study is primarily granted by the Science and Technology Plan Project’ of Guigang City (Guikegong No. 2021007).

## Conflict of Interest

The authors declare that the research was conducted in the absence of any commercial or financial relationships that could be construed as a potential conflict of interest.

## Publisher’s Note

All claims expressed in this article are solely those of the authors and do not necessarily represent those of their affiliated organizations, or those of the publisher, the editors and the reviewers. Any product that may be evaluated in this article, or claim that may be made by its manufacturer, is not guaranteed or endorsed by the publisher.
