# Supplementary figures and images for: Bioinformatics and In Silico Findings Uncover Bio-Targets of Calycosin Against Heart Failure and Diabetes Mellitus
Source: Front Endocrinol (Lausanne). 2022 Jul 8;13:790619. doi: 10.3389/fendo.2022.790619 (PMC9309256; doi:10.3389/fendo.2022.790619)

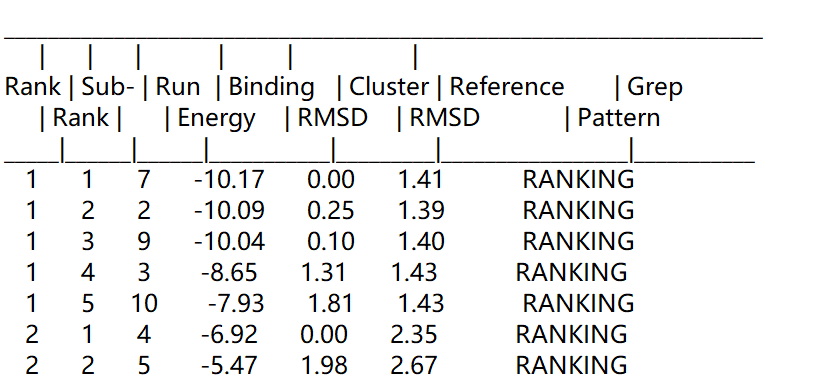

Supplement: Supplementary file 1 [file DataSheet_1.zip › Raw data/Docking/ABL1/RMSD 1.97.png]

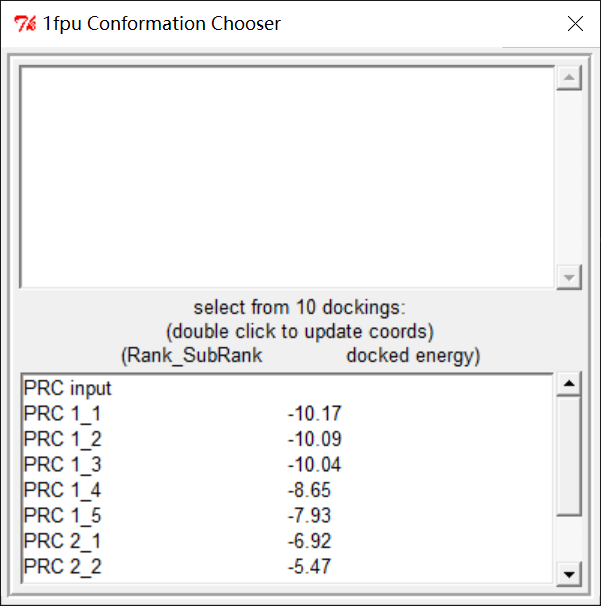

Supplement: Supplementary file 1 [file DataSheet_1.zip › Raw data/Docking/ABL1/╘¡╢╘╜╙─▄.png]

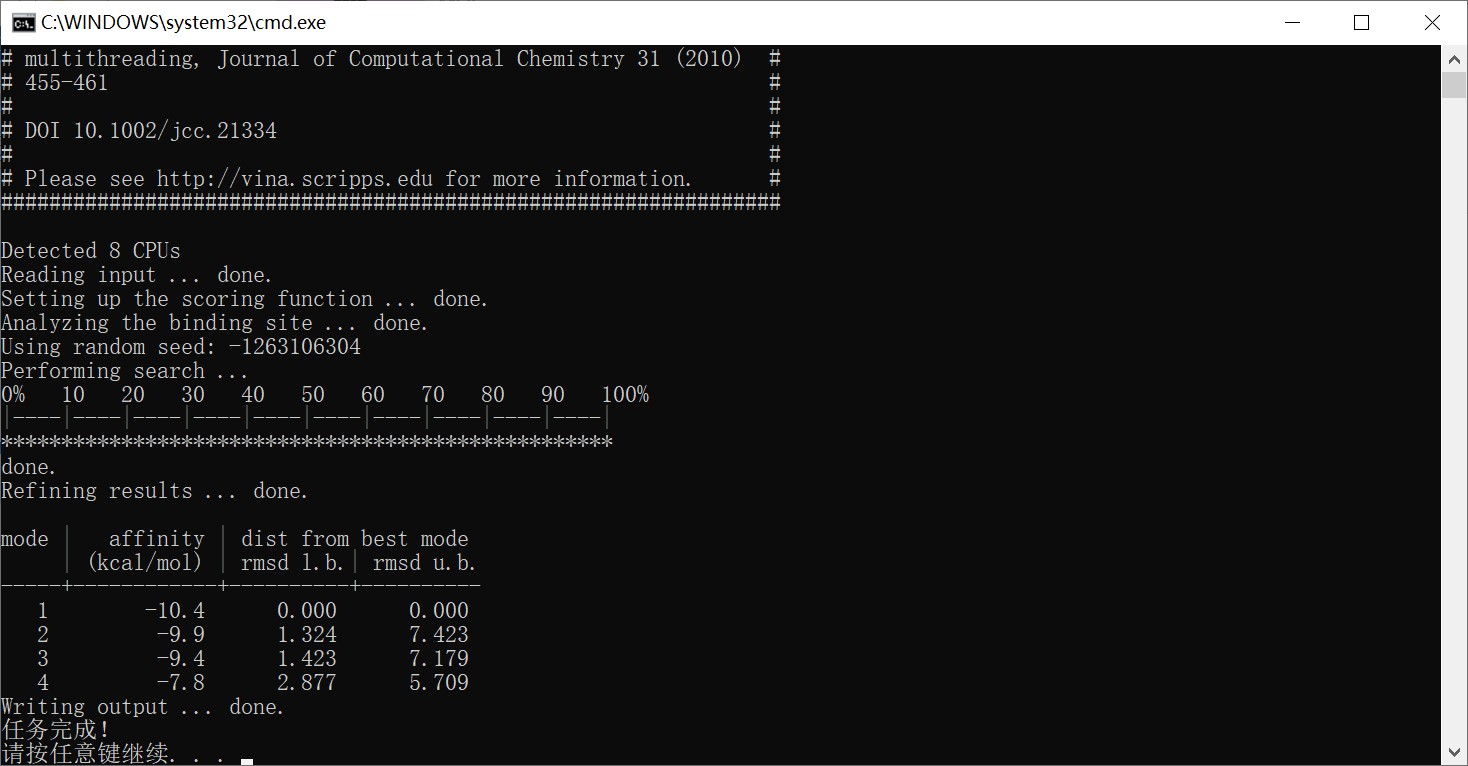

Supplement: Supplementary file 1 [file DataSheet_1.zip › Raw data/Docking/ABL1/╨┬╢╘╜╙─▄.png]

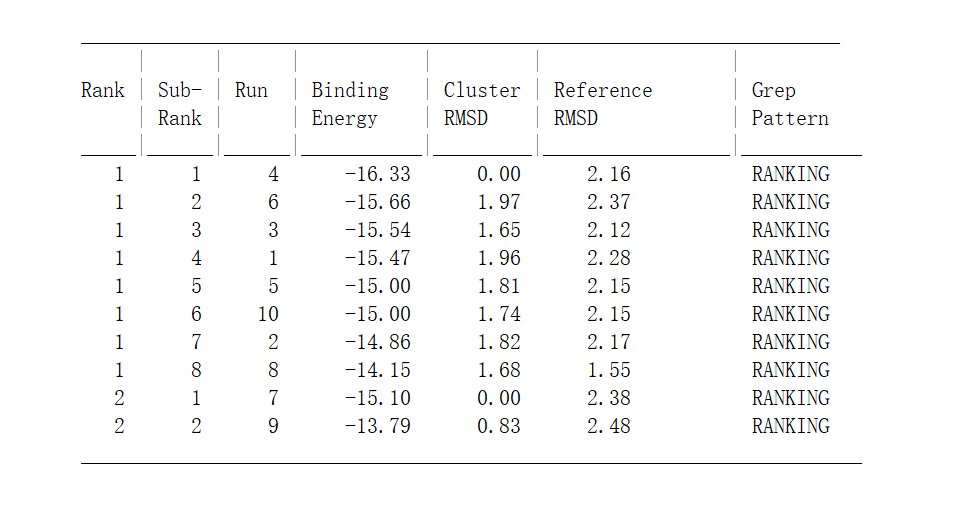

Supplement: Supplementary file 1 [file DataSheet_1.zip › Raw data/Docking/ARRB1/RMSD 2.18.png]

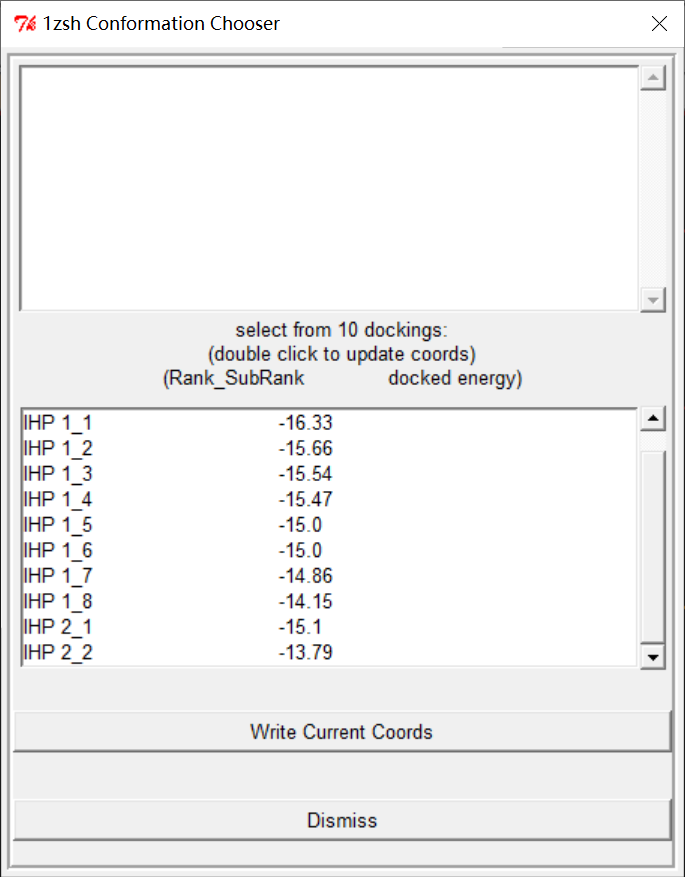

Supplement: Supplementary file 1 [file DataSheet_1.zip › Raw data/Docking/ARRB1/╘¡┼Σ╠σ╢╘╜┌─▄.png]

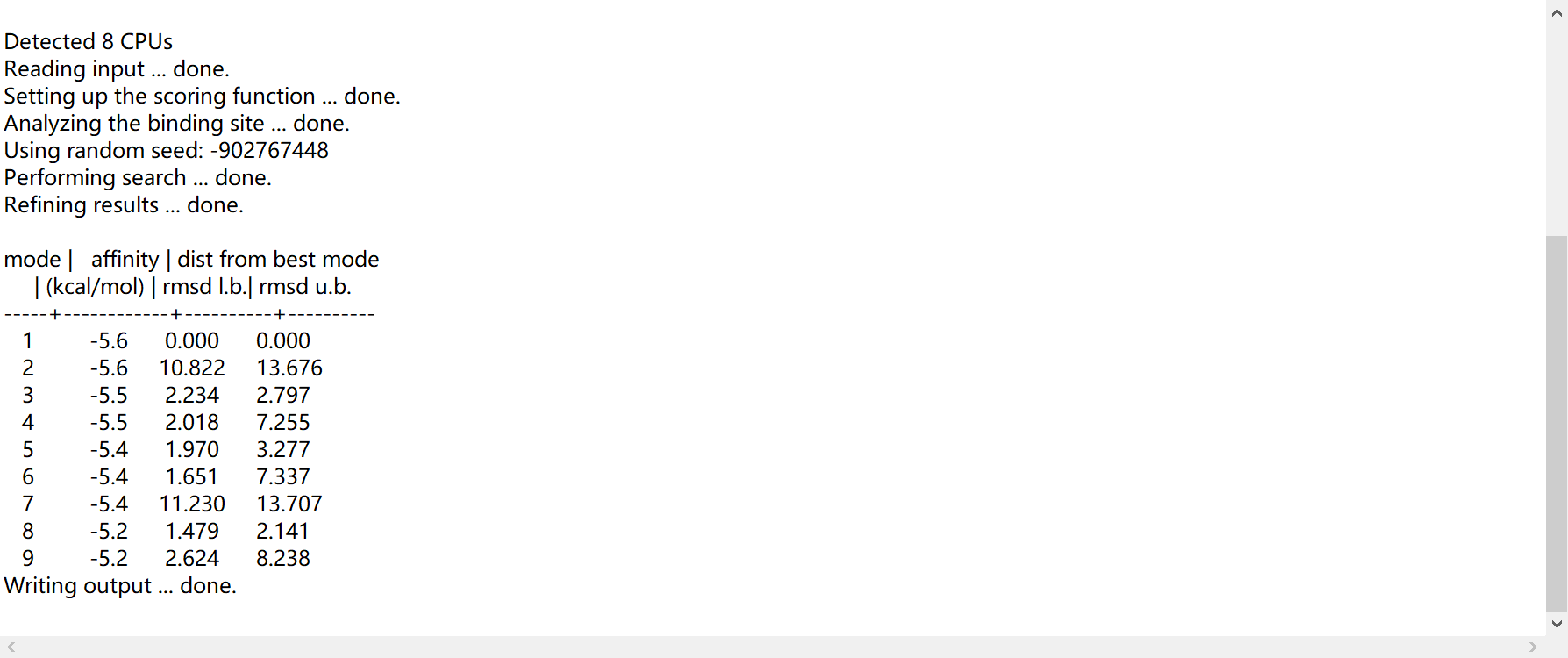

Supplement: Supplementary file 1 [file DataSheet_1.zip › Raw data/Docking/ARRB1/╨┬┼Σ╠σ╢╘╜╙─▄╢╘╜╙─▄.png]

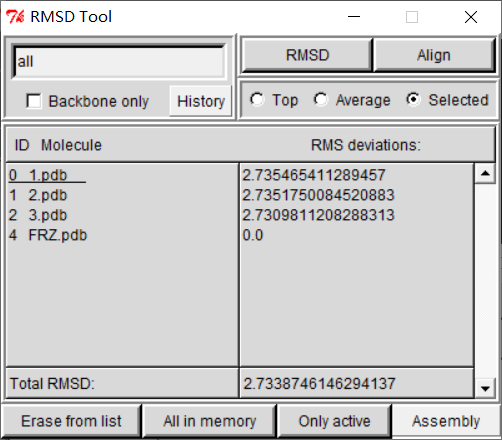

Supplement: Supplementary file 1 [file DataSheet_1.zip › Raw data/Docking/MAPK1/RMSD 2.73.png]

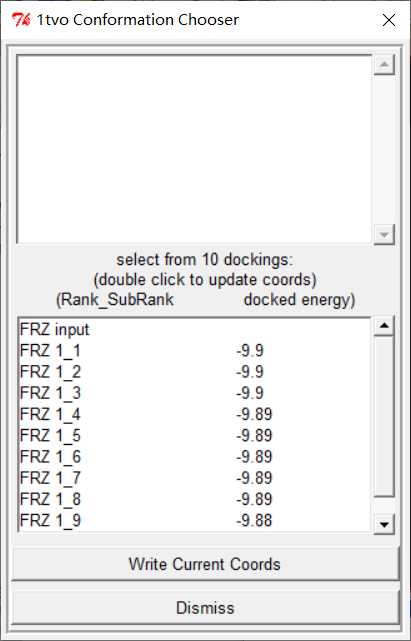

Supplement: Supplementary file 1 [file DataSheet_1.zip › Raw data/Docking/MAPK1/╘¡┼Σ╠σFRZ╢╘╜╙─▄.png]

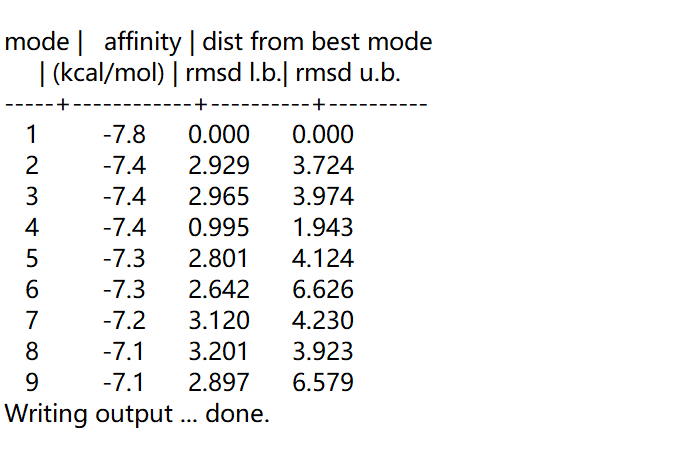

Supplement: Supplementary file 1 [file DataSheet_1.zip › Raw data/Docking/MAPK1/╨┬┼Σ╠σ╢╘╜╙─▄.png]
